# Molecular recognition by the KIX domain and its role in gene regulation

**DOI:** 10.1093/nar/gkt1147

**Published:** 2013-11-16

**Authors:** Jitendra K. Thakur, Archana Yadav, Gitanjali Yadav

**Affiliations:** National Institute of Plant Genome Research (NIPGR), Aruna Asaf Ali Marg, New Delhi 110067, India

## Abstract

The kinase-inducible domain interacting (KIX) domain is a highly conserved independently folding three-helix bundle that serves as a docking site for transcription factors, whereupon promoter activation and target specificity are achieved during gene regulation. This docking event is a harbinger of an intricate multi-protein assembly at the transcriptional apparatus and is regulated in a highly precise manner in view of the critical role it plays in multiple cellular processes. KIX domains have been characterized in transcriptional coactivators such as p300/CREB-binding protein and mediator of RNA polymerase II transcription subunit 15, and even recQ protein-like 5 helicases in various organisms. Their targets are often intrinsically disordered regions within the transactivation domains of transcription factors that attain stable secondary structure only upon complexation with KIX. In this article, we review the KIX domain in terms of its sequence and structure and present the various implications of its ability to act as a transcriptional switch, the mechanistic basis of molecular recognition by KIX, its binding specificity, target promiscuity, combinatorial potential and unique mode of regulation via allostery. We also discuss the possible roles of KIX domains in plants and hope that this review will accelerate scientific interest in KIX and pave the way for novel avenues of research on this critical domain.

## INTRODUCTION

**Figure 1.**
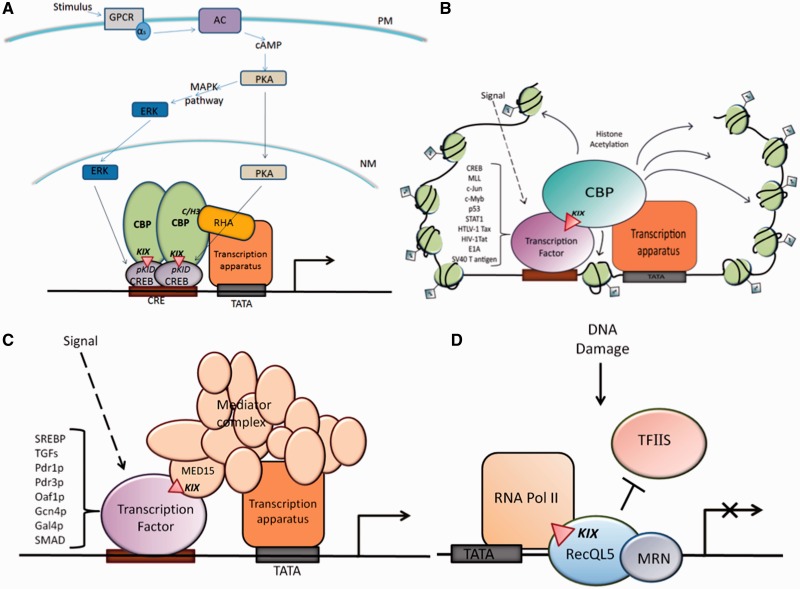
Cartoon depicting the varied interactions and overall role/s played by KIX in **(A)** the general transcriptional apparatus, **(B)** regulation via CBP and its coactivators, **(C)** the mediator complex and **(D)** the RecQL5 helicase.

Transcriptional activation is a key process in the regulation of gene expression in living organisms, and more so in eukaryotes where the transcriptional apparatus is a huge modular complex. Its regulation in eukaryotes is accomplished through an intricate network of specific interactions at the DNA–protein interface and the protein–protein interface. Transcriptional activators achieve gene specificity by binding specific regulatory elements on DNA via functionally independent DNA-binding domains. In addition, transcription factors mediate recruitment of other factors, subsequent chromatin modification and pre-initiation complex formation by docking one or more of their transactivation domains to conserved sites on many cognate proteins, like, for example, transcriptional coactivators that are important binding partners for activation domains (ADs). These coactivators thus serve as bridging molecules between transcription factors and the transcription machinery, or as enzymes that introduce chemical modifications on DNA. One such coactivator is the cAMP-response element-binding protein (CREB)-binding protein (CBP), a large multi-domain protein containing stretches of intrinsically disordered linker regions and a number of structured protein-binding domains that interact with a large variety of transcription factors. One of the domains of CBP is a vital, independently folding and highly conserved three-helix bundle region termed as the kinase-inducible domain interacting (KIX) domain. It was discovered by Parker *et al.* (1), in 1996 in the mouse CBP, as the specific and minimal region that was sufficient to bind and interact with phosphorylated CREB and then activate transcription. Since its initial identification was based on its interaction with the phosphorylated kinase-inducible domain (KID) region of CREB protein, it was given the name ‘KID-interacting domain’ or ‘KIX’ (1). Following its discovery in CBP, KIX domain has been identified in the human activator recruited cofactor 105-kDa component (ARC105) and yeast Gal11p both of which are mediator of RNA polymerase II (RNA Pol II) transcription subunit 15 (MED15) subunit of mediator complex involved in regulation of transcription of specific genes (2–4). As shown in Figure 1, KIX is now known to be one of the most important molecular recognition sites for protein–protein interaction during gene regulation, playing a significant role in assembly of proteins to the transcriptional apparatus in yeast and mammals (1,2,5). KIX has been shown to interact with a range of transcription factors other than CREB such as c-Myb (6), mixed lineage leukemia protein (MLL) (7), breast cancer 1 (BRCA1) (8), c-Jun (9), p53 (10), signal transducers and activators of transcription (STAT1) (11) and sterol responsive element-binding protein (SREBP) (12) of humans (Figure 1A–C) to Gal4p (13), Gcn4 (14), Pdr1/3 (2) and Oaf1 (3) of yeast (Figure 1C), cubitus interruptus (15) of *Drosophila* and human t-lymphotropic virus type 1 (HTLV-1) Tax (16), human immunodeficiency virus type 1 (HIV-1) Tat (17) and E2 (18) of viruses (Figure 1B). Direct involvement of KIX domain has been demonstrated in long term storage of hippocampus-dependent memory in mammalian brains (19), lipid homeostasis in mammals (4), multi-drug resistance (2) and fatty acid metabolism (3) in yeast, and salicylic acid mediated defense response in *Arabidopsis* (20). Importance of KIX domain in determination of seed size of rice has been postulated by presence of single nucleotide polymorphisms (SNPs) differentiating the genotypes of long grain from that of short grain (21). KIX domain has recently been reported from human recQ protein-like 5 (RECQL5) helicase as an RNA Pol II-binding domain (Figure 1D), which is in contrast to the regular role of KIX domains of CBP, ARC105 and Gal11p that bind to ADs of transcription activators (2–4,22). In this case, the KIX domain of RECQL5 interacts with Rpb1 jaw domain of RNA Pol II to repress transcription (22). Thus, the proteins that possess KIX domain namely CBP, mediator subunit MED15 and most recently helicase RecQL5 are mainly transcriptional regulators, and the major role of KIX is to act as a docking site for transcription factors and for stabilization of protein–protein interactions (1,2,5,23,24). Solution structures of several KIX domains in mouse, humans and yeast are available in the protein data bank (PDB), both in ligand-bound complexes and in the unbound forms. According to the Pfam database, KIX domain also shows conservation in the plant kingdom and other groups such as arthropods and nematodes, although the structure of KIX in these groups is not yet characterized.

Despite elucidation of its crucial role in transcription, several gaps remain in our knowledge of the KIX domain, specifically in terms of its ability to recognize and assist in folding of intrinsically disordered proteins, its role in secondary structure determination, its varying binding modes, mechanism of allostery, high structural plasticity and even some of its cellular functions. Furthermore, very little is known about KIX domain proteins in plants, although a recent association analysis of SNP loci in 23 rice genotypes identified three non-synonymous SNP loci in plant KIX domains that showed strong association with important agronomic traits like grain size (21). Thus, it is particularly relevant to review the KIX domain across various life forms and integrate the contributions of recent studies in context of earlier reports to gain insights into the precise nature of its interactions and functions, and its potential for combinatorial targeting and allosteric regulation in diverse processes such as cell cycle, multi-drug resistance and fatty acid metabolism. In this review, we explore the sequence, structure, binding modes, docking plasticity and mechanistic aspects of KIX and its role in formation and stabilization of vital multi-protein complexes during transcriptional activation. We hope that the present review will open up multiple new avenues for further research on this important domain.

## DISCOVERY OF KIX AND SIGNIFICANCE OF THE CREB–CBP COMPLEX

The KIX was discovered as a stretch of ∼90 residues in mouse CBP that behaved as an inducible high affinity docking site for phosphorylated *Ser-133* of CREB KID domain, resulting in CREB–CBP complex formation (1). Although many other KIX domains have been characterized since then, along with their transcription factors, this first reported interaction between CBP KIX and CREB KID of mouse, resulting in the CREB–CBP complex, remains the most extensively studied and well understood in molecular terms. CREB is a well-known transcription factor, which in response to cAMP regulates cellular gene expression by binding to cAMP response element (CRE). CRE (5′-TGACGTCA-3′) is a conserved region present in promoters of many genes and is recognized by the CREB dimer (25). As shown in Figure 1A, protein kinases activate CREB by phosphorylating a particular conserved serine residue in its KID domain when external stimuli such as calcium, growth factors, protein hormones, light etc., triggers signaling cascades (1,26–28). As can be seen in this figure, CBP–CREB complexation occurs via KID–KIX interaction, and this leads to interaction of CBP with RNA Pol II at the cysteine and histidine rich domain 3 (C/H3) of CBP domain site, thereby activating transcription (29). There have been suggestions that CBP interacts with transcription factor II B as well as with TATA-binding protein, a component of transcription factor II D (30,31). Although CBP and p300 are present in limited amount in nucleus, they are known to associate with large variety of proteins most of which are transcription factors such as CREB, STAT1, p53, c-Myb etc., as shown in Figure 1B.CBP/p300 proteins perform crucial roles in various cellular events because CBP/p300 is a common target for many transcription factors, which are involved in cell growth, differentiation, proliferation, repair and apoptosis (32–34). Furthermore, CBP is a target of many viral oncoproteins such as HTLV-1 TAX, adenovirus early region 1A (E1A) and simian vacuolating virus 40 T(SV40-T) antigen (Figure 1B). E1A is an antagonist to p300/CBP-associated factor (pCAF), which binds CBP/p300 during cell cycle. The CBP/p300-pCAF complex can arrest cell cycle progression and E1A targets CBP/p300 and drives cell into S phase to cause tumors (35). Thus CBP not only acts as a molecular bridge that stabilizes the transcription apparatus but also tightly regulates the expression of specific genes when recruited by different transcription factors. It does so by using distinct domains within its sequence namely receptor interaction domain, KIX and transcription adaptor putative zinc finger, all of which enable it to interact with such a wide range of transcription factors. CBP/p300 also has an intrinsic histone acetyltransferase (HAT) activity that allows it to activate transcription through chromatin remodeling and alteration of DNA accessibility (Figure 1B), apart from playing a critical role in nuclear receptor (NR) signaling (36,37).

## ROLE OF KIX IN COMPLEXATION AND STRUCTURAL STABILIZATION

**Figure 2.**
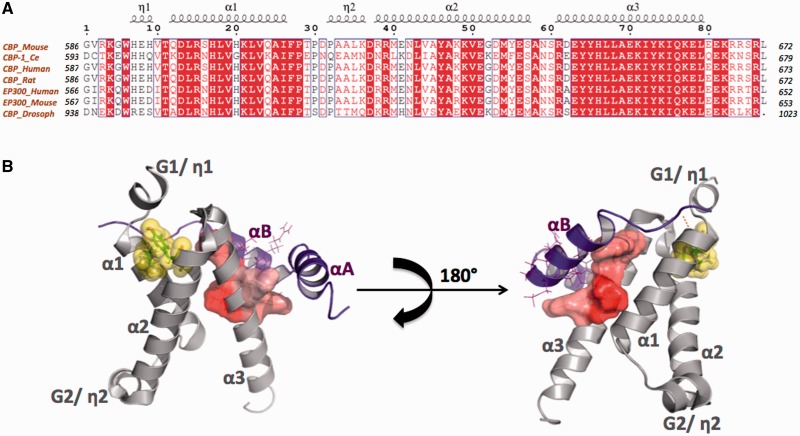
The CBP-KIX domain sequence and structure. **(A)** Alignment of selected KIX domain sequences showing high conservation of all secondary structural elements. **(B)** Opposing views of the KIX domain structure (gray) in complex with pKID (purple), depicting the helical nature of both proteins in cartoon representation (PDB ID 2LXT). Secondary structural elements are marked in gray and purple for KIX and pKID, respectively. The highly hydrophobic surface that binds KID is shown in shaded red representation. The cation–pi interaction between the aromatic ring of Tyr-640 and guanidinium group of Arg-300 that stabilizes the KIX domain structure is depicted in yellow.

The intimate physical binding of CREB and CBP requires certain domains from both sides. This is where the role of KIX domain comes into play, with KIX being the specific site in CBP that binds to phosphorylated KID (pKID) region of CREB, as shown in Figure 1A.The CBP-KIX domain sequence, comprising ∼90 amino acids, is highly conserved in yeast and animals. Figure 2A depicts the alignment of selected CBP-KIX domains. At the time of the discovery of mouse CBP KIX domain, circular dichroism studies predicted it to be rich in alpha helices and hydrophobic interactions were found to play a dominating role in complex formation (1). This was confirmed by the first nuclear magnetic resonance (NMR) structure of KIX revealing it to be a small bundle of three mutually interacting alpha helices and two short 3_10_ helices joined by small loops enclosing a hydrophobic region as shown in Figure 2B (38). As per convention, the alpha helices of KIX domain are denoted as α1, α2 and α3 while 3_10_ helices as G1 and G2. As can be seen in Figure 2B, helices α1 and α3 helices are coplanar and also approximately parallel to one another at an angle of ∼17°, whereas helices α1 and α2 are at an angle of ∼55° to one another. Residues of α1 and α2 share contacts throughout their lengths, whereas residue contacts between α2 and α3 are very limited. The first 3_10_ helix, G1 lies near N terminus of KIX domain, whereas the second 3_10_ helix, G2, lies in the linker region between α1 and α2, which is more flexible and longer than linker region between α2 and α3 (Figure 2B).

**Figure 3.**
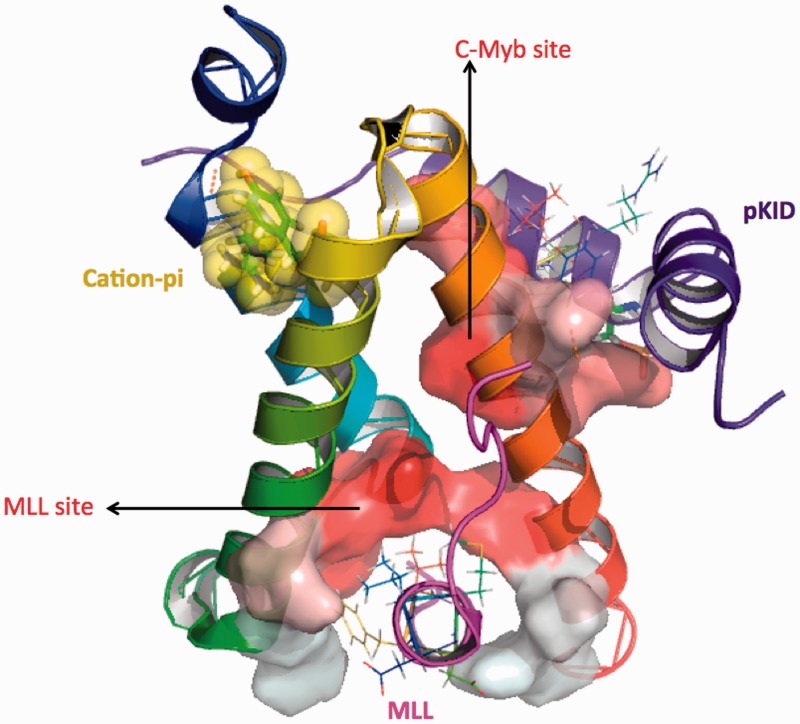
3D view of the ternary complex of KIX domain (PDB ID 2LXT) depicting two distinct, spatially separated binding surfaces. Each binding surface is colored by hydrophobicity (graded scale of red) and lies on opposite sides of the KIX domain, represented in cartoon with rainbow colors. The two binding partners, MLL (magenta) and KID (purple) are also shown at their respective sites on KIX. The TAD motifs in both ligands are depicted as lines. The cation–pi interaction is shown in yellow surface representation.

KIX plays a dominant role in secondary structure formation of pKID because the isolated KID region is largely disordered and devoid of significant preformed helicity. After binding to KIX, the pKID adopts an ordered structure of two distinct mutually perpendicular helices (namely αA and αB) linked by a turn, that wrap around α3 of KIX in a shallow hydrophobic pocket on KIX surface (Figure 2B). This pKID–KIX interaction is stabilized both by hydrophobic and electrostatic interactions, the former betweenαB of KID and the KIX hydrophobic pocket in the region of helices α1 and α3, and the latter between specific charged residues on each domain. It is interesting that both αA and αB of pKID are mainly stabilized by KIX domain’s interacting surface and intra pKID interactions do not play any important role in its stabilization (38). The core globular KIX domain is stabilized by a cation–pi interaction between aromatic ring of *Tyr-640* and positively charged guanidinium group of a highly conserved *Arg-*600 (Figure 2B). It has been shown that replacement of this arginine by glutamine can impair KIX binding even though *Arg-600* does not make direct contacts with KID of CREB (1). Interestingly, since the cation–pi interaction is considered crucial for specifying the folded KIX structure (Figure 3), disruption of this interaction by arginine methylation may serve as a regulatory mechanism of inhibition of KIX function, and is discussed in the penultimate section.

Apart from the cation–pi interaction, residues that lie beyond the globular KIX core have been shown to influence its secondary structure and stability. In particular, a stretch of six residues (KRRSRL) observed at the C terminus of an extended α3 helix in some KIX domains (see alignment in Figure 2A), has been shown to be important for stabilization. Since these residues are not part of the hydrophobic core of KIX, they have been proposed to contribute indirectly to helix stabilization via electrostatic interactions with the helix dipole, based on chemical shift perturbation mapping (39).

The phosphorylation event is also critical for stability of the pKID–KIX complex since the phosphorylated serine residue in CREB is directly involved in the binding event with CBP/p300. In human CREB, phosphorylation site of KID is *Ser-118*, whereas it is 133 in rat and mouse CREB. *pSer-133* is essential for complex formation as its mutation diminishes KID–KIX affinity. This residue is present in the same helix (αB) of KID that makes direct contacts with the KIX domain. The phosphate group of *pSer-133* forms a hydrogen bond with *Tyr-658* and a salt bridge with *Lys-662* of KIX domain (38,40). This coupled binding and folding event introduces a large entropy barrier, which is compensated by the electrostatic contribution of the phosphate group to the binding free energy, thereby making this phosphorylation event a critical thermodynamic switch for the inducible formation of the CREB–CBP complex (41). Furthermore, such folding after binding facilitates selectivity and speed of interaction, and also lets the CREB exclusively interact with CBP among other coactivators (18,42). Two critical points regarding KIX may be noted here; first, KIX binding is not always phosphorylation dependent as in the case of c-Myb, an oncoprotein essential for cellular proliferations of immature hematopoietic cells, that constitutively interacts with CBP at its KIX domain (43). Since interaction between c-Myb and KIX is not phosphorylation dependent, contributions to hydrogen bond and salt bridge formation from *Tyr-658* and *Lys-662* of KIX were not observed. Interestingly this may account, in large part, for the ∼50-folds lower affinity of interaction between c-Myb and CBP as compared to that between CREB-KID and CBP, detected by fluorescence anisotropy and ITC experiments (44,45). Second, not all protein kinases can result in KID–KIX complexation, making this interaction stimulus specific. For example, Ca^2+^/calmodulin-dependent protein kinase II is incapable of stimulating CREB activity although it can phosphorylate *Ser-133* of KID. This is because certain protein kinases additionally phosphorylate *Ser-142* in αB of pKID and phosphorylation at *Ser-142* has an inhibitory effect on complexation (46). Phosphorylated *Ser-142* causes juxtaposition of a bulky group near *Tyr-650*, which in turn weakens the interaction between αB of pKID and hydrophobic groove of KIX, thereby blocking formation of KIX–KID, and further implications are discussed in a later section (40,46).

## CONSERVATION OF KIX IN THE MEDIATOR SUBUNIT MED15

Apart from CBP/p300, homologous KIX domains have also been identified and characterized in MED15, a subunit of the mediator complex found in all eukaryotes. Functional and structural studies have revealed significant similarity between KIX domains of MED15 proteins of diverse organisms, at sequence, structure and functional levels. The KIX domain in MED15 (also called ARC105), a subunit in the mediator/ARC complex of humans, was originally identified as a SREBP1a-binding domain (5,12). SREBP proteins belong to a family of transcription factors involved in cholesterol and lipid homeostasis in mammals, and they bind to a region on ARC105 with significant sequence and structural similarity with the CBP KIX domain (4). SREBP1a has been shown to act as a common substrate for both CBP KIX and ARC105 KIX, although it is not the same with some other transcriptional activators such as CREB and c-Myb, which do not bind significantly with ARC105 KIX domain, implying an unexpected specificity among KIX domains (4). This specificity can potentially be ascribed to the absence of residues *Tyr-658* and *Lys-662* in MED15, since both are present in α3 of CBP KIX and have been implicated in the interaction with protein kinase A-phosphorylated CREB and c-Myb ADs (40,45). Thus, it is the residues in helix α3 of KIX that provide activator binding specificity, and the lack of conservation of these residues in ARC105 KIX is the reason why CREB and c-Myb are unable to effectively bind to ARC105 KIX domain (4).

Gal11p/MED15 is a subunit of mediator complex in yeast that also harbors KIX domain, initially discovered as Pdr1p-binding domain with a specific role in xenobiotic-dependent regulation of multidrug resistance in yeast and pathogenic fungi (2). Family members of Pdr1/3p in *Saccharomyces cerevisiae* and *Candida glabrata* recognize the presence of xenobiotics by directly binding to them and then binding to KIX domain of Gal11p (2,47). KIX domain of Gal11p/MED15 interacts with ADs of several transcription factors such as Gal4p, Pdr1/Pdr3p, Oaf1p, Gcn4p, etc. (2,3,13,14). NMR studies indicated substantial structural similarity of this domain to human ARC105/MED15 KIX domain, and there was evidence of a similar hydrophobic core in both proteins forming the binding interface for various transcription factors. This resemblance to human MED15 KIX domain was found to extend in functional terms as well, since Gal11p KIX domain could interact with human SREBP1a, but not with CREB and c-Myb activators (2). Gal11p KIX domain also plays an important role in the regulation of lipid metabolism in fungi, where fatty acids such as oleic acid (OA), lauric acid (LA), myristic acid (MA) and palmitic acid (PA) are able to directly bind to transcription factor Oaf1p and activate it to induce expression of Fatty Acid oxidation 2 (FOX2) and FOX3 genes that are involved in metabolism (3). Here too, the Gal11p KIX domain was found to directly interact with Oaf1p, at a hydrophobic groove formed between three α helices (with α2 participating in the majority of interactions), resulting in recruitment of the transcriptional machinery. This activation, however, was specific to Oaf1 interaction with OA, LA, PA and MA, but not stearic acid for instance, or other molecules like peroxisome proliferators or non-steroidal anti-inflammatory drugs that could bind to Oaf1p, but could not stimulate Oaf1–KIX interaction or consequently, transcriptional activation, suggesting that specific conformational changes are facilitated in Oaf1 by cognate ligands that stimulate KIX–Oaf1 interaction (3).

## KIX DOMAIN IN RECQL5 AND ITS ROLE IN STRUCTURAL MIMICRY

The most recent KIX domain has been reported from RecQL5, one of the five human DNA helicases employed for opening the replication fork, and this report led to the discovery of a vital function played by KIX domain in DNA repair during replication (23). The RecQL5 helicase is known to be essential for reducing tumor risk and genome stabilization during transcription, replication, recombination and DNA repair. It is the only human RecQ helicase that can interact directly with RNA Pol II (48). Computational modeling revealed a KIX like region in RecQL5 that resembled the KIX domains in CBP, p300 and MED15 in terms of sequence and the common three-helix bundle structure, with a conserved hydrophobic core as well. Mutations in this region of RecQL5 led to disruption of interaction between RecQL5 and RNA Pol II, which in turn led to inhibition of RNA Pol II during initiation and elongation events of transcription, suggesting a crucial and specific role of KIX in transcriptional regulation, as shown in Figure 1D (23). Later, it was shown that RecQL5 helicase could recognize double strand breaks during DNA damage and stall further transcription by interacting with, and inhibiting RNA Pol II through its KIX domain, thereby enabling cell survival during DNA damage (49). A very recent structural investigation into the mechanistic basis of this inhibition revealed a surprising structural overlap between RecQL5 KIX domain and domain II of elongation factor TFIIS, suggesting a dual mode of transcriptional repression wherein the KIX domain enables RecQL5 to structurally mimic the Pol II–TFIIS interaction, thereby occluding the TFIIS-binding site on Pol II, which in turn prevents transcription from resuming. The RecQL5 KIX domain and domain II of yeast and metazoan TFIIS orthologs show sequence conservation and the authors employed three-dimensional (3D) crystallography to illustrate structural mimicry wherein the KIX domain assumes a TFIIS like structure and binds to Pol II in a region that overlaps with its known binding site for TFIIS (22). A large number of cellular signals necessitate short-term suspension of the transcriptional machinery and this mechanism of repression via blocking of TFIIS normal function might operate as a general mode of response and regulation in the cell.

## KIX PROMISCUITY: MULTIPLE BINDING SITES

For several years post-discovery, the KIX domain was assumed to have a single binding site, despite observations that certain viral transcriptional activators like HTLV1-Tax and p53 could interact with the CREB-bound form of KIX (50–52). These interactions were believed to be mutually exclusive and the initial solution structures of KIX in complex with KID and c-Myb also supported the single-site notion, since both KID and c-Myb were observed to share a common binding site despite the fact that KID and c-Myb are quite distinct from each other in critical aspects of KIX mediated interaction. These include the facts that, (i) the KIX-binding region of c-Myb has no obvious sequence similarity with the equivalent region on KID, (ii) c-Myb binds CBP in a phosphorylation independent manner unlike KID (45) and (iii) the affinity of KIX for pKID is 20-fold higher than that for c-Myb (53). Furthermore, initial CD- and NMR-based studies had suggested that despite both being intrinsically unstructured or disordered proteins (IDPs), c-Myb is already in partial (∼25%) helical conformation in unbound state unlike KID which has only 1% helicity when unbound (44,45).

In 2002, two independent groups working with MLL and c-Jun coactivators discovered a secondary binding site in KIX through NMR chemical shift mapping, a site that was remote and distinct from the one employed by KID/c-Myb (7,54). MLL is crucial for production of normal blood cells and is a homolog of the *Drosophila* trithorax (Trx) protein involved in maintenance of homeobox (Hox) gene expression during early embryogenesis, while c-Jun is a human transcriptional coactivator and a mediator of protein–protein interactions, involved in diverse cellular process including differentiation and apoptosis. The first discovery was the outcome of an investigation into whether MLL and c-Myb proteins were competing for CBP binding or instead interacting cooperatively with CBP, based on the facts that, (i) MLL is a binding partner for both c-Myb and pKID, (ii) both MLL and c-Myb were known to express at the same time during development of hematopoietic precursor cells, (iii) both play a pivotal role in blood cell proliferation, and finally, (iv) both could bind KIX domain (7,55–58). Goto *et al.* (7), found evidence for cooperativity, as occupation of one binding site on KIX decreased the entropic penalty associated with the second binding event, and while localizing the MLL-binding site on KIX, spectral differences indicated binding to a remote and new site. Similarly, in case of c-Jun, the chemical shift perturbations arising from conformational changes of aromatic rings induced by pJun were found to be smaller than those induced by pKID, revealing higher aromaticity in vicinity of the pKID–KIX complex (*Tyr-640*, *Tyr-649*, *Trp-591* and *Tyr-658* of KIX, and *Tyr-134* of pKID) in contrast to a single aromatic side chain *Tyr-631* of KIX in the vicinity of the c-Jun-binding site revealing two distinct interaction surfaces (54). These initial reports showed that both ligands (c-Myb and c-Jun/MLL) could bind simultaneously and cooperatively to the CBP KIX domain in their corresponding hydrophobic grooves making ternary complexes, providing a role for KIX in transcriptional synergy. Figure 3 depicts the ternary complex of KIX with KID and MLL, and it can be seen that the KID/c-Myb site consists of a hydrophobic groove formed by α1 and α3 helices of KIX whereas the second, spatially separated MLL site is on the opposite surface of KIX and consists of a groove formed at the interface between G2, α1 and α3 helices of KIX domain. Later studies firmly established the presence of two independent binding surfaces on CBP KIX domain, designated as c-Myb and MLL sites (55,59). MLL site has since been shown to bind many other proteins, including c-Jun, HTLV-1 oncoprotein TAX and HIV-1 transactivator of transcription (TAT) (17,60). Table 1 provides a list of various transcription factors that bind to these two binding sites.

**Table 1.** Details of known binding partners for the two different binding surfaces on the KIX domains of CBP

S.no	Interacting protein	Interacting domain	Interaction site on CBP/p300 KIX	Phosphorylation dependence	Reference
1	CREB	KID	c-Myb site	+	(38)
2	c-Myb	TAD	c-Myb site	−	(45)
3	p53	TAD1 and TAD2	MLL, c-Myb site	+	(61)
4	MLL	TAD	MLL	−	(59)
5	FOXO3a	CR2C and CR3	MLL, c-Myb site	+	(62)
6	TAX	AD	MLL site	−	(60)
7	c-Jun	AD	MLL site	−	(54)
8	BRCA1	BRCT domain	c-Myb site	−	(63)
9	SREBP	AD	C-terminal of α3	−	(4)
10	HIV-1 TAT	AD	MLL	−	(17)

KIX thus provides two distinct binding surfaces for interactions with transcriptional ADs and it is interesting to note that both binding sites interact with peptides that contain the characteristic amphipathic φ-x-x-φ-φ motif (φ: bulky hydrophobic residue and x: any residue). This conserved TAD motif does not provide any binding site specificity to transcription factors as it is observed in the amphipathic α helices of transactivation domains of many transcription factors such as CREB-KID, MLL, c-Jun, c-Myb, p53, E2A and Forkhead box class O 3a (FOXO3a) (64). This similarity of TAD motif recognition between the two sites extends in terms of function as well, and combined with binding cooperativity, it provides new insights into the combinatorial recruitment of CBP by multiple ADs. The simultaneous binding pattern combined with limiting quantities of CBP in cells suggest KIX to be a potential link between diverse transcription factors contributing significantly to the synergy of gene expression, and resulting in enhanced recruitment of CBP to target promoters.

Interestingly, as mentioned in Table 1, some transcription factors like p53 and FOXO3a have the ability to bind both the sites of KIX domain by means of two tandem ADs within their sequences, and both transactivation domains can simultaneously acquire the KID and MLL sites of KIX (61,62). FOXO3a is very important since it activates transcription of genes involved in cell differentiation, DNA repair, apoptosis and stress response. It is an intrinsically disordered protein with three conserved regions (CR1–CR3), of which the CR3 and the C-terminal segment of conserved region 2 (CR2C) are both TADs that can each occupy both the c-Myb and MLL sites through multiple promiscuous and dynamic interactions, resulting in two stable conformations. Similarly, the tumor suppressor p53, which causes cell arrest and apoptosis during DNA damage, also has two transactivation domains AD1 and AD2, and KIX acts as a sensor of phosphorylation state of these two TADs, thereby mediating cell cycle regulation. In contrast to FOXO3a, the tandem p53 TADs can both bind in two different orientations to each of the two KIX sites, thereby supporting eight distinct binding modes. Studies have shown that unlike other transcription factors, AD1 and AD2 transactivation domains of p53 have similar affinity to bind both pKID/c-Myb and MLL sites of KIX domain while AD1 binds preferentially to MLL site (65). The binding is also phosphorylation dependent like that of pKID although phosphorylated AD1 binds preferentially to MLL site of KIX domain (61). Overall, the MLL site exhibits remarkable plasticity being able to accommodate a variety of peptides in various orientations without any substantial structural rearrangement (62).

It is noteworthy that despite its small size and a relatively simple helical bundle fold, KIX has two distinct sites, both with the ability to bind different IDP systems. For a long time, it was not possible to exactly unveil the mechanism of this binding since, dissecting the mechanism of recognition between IDPs and their physiological partners can be extremely complex and requires extensive kinetic characterization. However, such a study has recently been conducted despite the inherent complexity of the process, resulting in the establishment of a unique ‘binding-precedes-folding’ mechanism for KIX binding to c-Myb. The authors clearly demonstrate that the transactivation domain of c-Myb recognizes KIX domain in its unstructured conformation and binds to it via a salt bridge between *Arg-61* and *Glu-306*, subsequently followed by a rapid folding step driven by h-bond interactions at the opposite side of the interface (66). It is presumable that the mechanism of interaction between KIX and other IDPs may display conserved features of a similar kind.

## THE ROLE OF KIX IN ALLOSTERY

KIX plays a crucial role from a regulatory perspective also, since it has the capability of binding the same transcription factors in structurally distinct modes, in addition to the capacity of binding two different transcription factors at the same time. By virtue of spatially separated binding sites, KIX can directly mediate interactions between bound transcription factors, acting as a bridge between p53 and TAX or CREB and TAT. The previously discussed synergistic effect between the two KIX-binding sites, i.e. effect on binding of a transcription factor to one site of CBP-KIX domain in the presence of another transcription factor already bound to the second site, also promotes allosteric regulation. Table 2 provides a list of interactions that have been observed and reported to be involved in cooperative binding in KIX to date. Investigations into thermodynamics of this cooperativity using isothermal titration calorimetry have shown that second binding event is accompanied by a 2-fold enhancement in binding affinity (7). This holds true in both directions, i.e. binding of MLL to KIX enhances the binding of c-Myb at pKID site of KIX domain by ∼2-fold higher affinity, and conversely, binding of c-Myb to KIX enhances the MLL binding affinity to KIX, whereupon the ternary complex activates expression of various downstream genes (7,59,67). The same holds true for the ternary complex involving pKID instead of c-Myb, although the mechanism of stabilization is different, suggesting a role for the KIX-binding sites as allosteric sites that undergo conformational changes leading to increased affinity with the second ligand. These conformational changes can be of two kinds. First, as observed for the ternary complex of KIX:c-Myb:MLL, the favorable enthalpy associated with binding of the second ligand to KIX domain decreases when the complementary ligand occupies the first binding site, implying an increase in the number or stability of electrostatic or polar interactions in the ternary complex. Alternatively, the observed cooperativity can arise on account of an increase in the favorable entropy upon ternary complex formation, implying a contribution of hydrophobic interactions, as observed for the ternary complex of KIX:pKID:MLL (7). Other proteins like HTLV-1, HTLV-1 basic leucine zipper factor (HBZ) and CREB-KID make ternary complexes when bound to KIX domain and both phosphorylated CREB and HBZ interact with KIX domain of CBP/p300 simultaneously at different binding sites and activate HTLV1 transcription (64). TAX also uses the same site as that of HBZ to bind KIX domain of CBP and to form ternary complex with CREB (60,68).

**Table 2.** Examples of cooperative binding in KIX

Cooperative binding	Cooperative effect
MLL-KIX-c-Myb	Binding of MLL to KIX increases the affinity of KIX for c-Myb and thus facilitates normal blood cell development.
HBZ-KIX-c-Myb	HBZ interaction with KIX causes disruption of cellular transcription by acting as competitive inhibitor to MLL. It enhances the binding of c-Myb-AD to KIX and thus leads to continued proliferation of leukemia cells.
HBZ-KIX-pKID	HBZ interaction with KIX causes enhanced binding of pKID to KIX and unregulated cell proliferation causing T-cell leukemia.
MLL-KIX-pKID	MLL–KIX interaction promotes favorable conformational rearrangements and enhanced binding of pKID to KIX thus increased activation of CREB responsive genes.
TAX-KIX-pKID	TAX interaction with KIX causes activation of HTLV-1 mediated genes thus causing T-cell leukemia.

**Figure 4.**
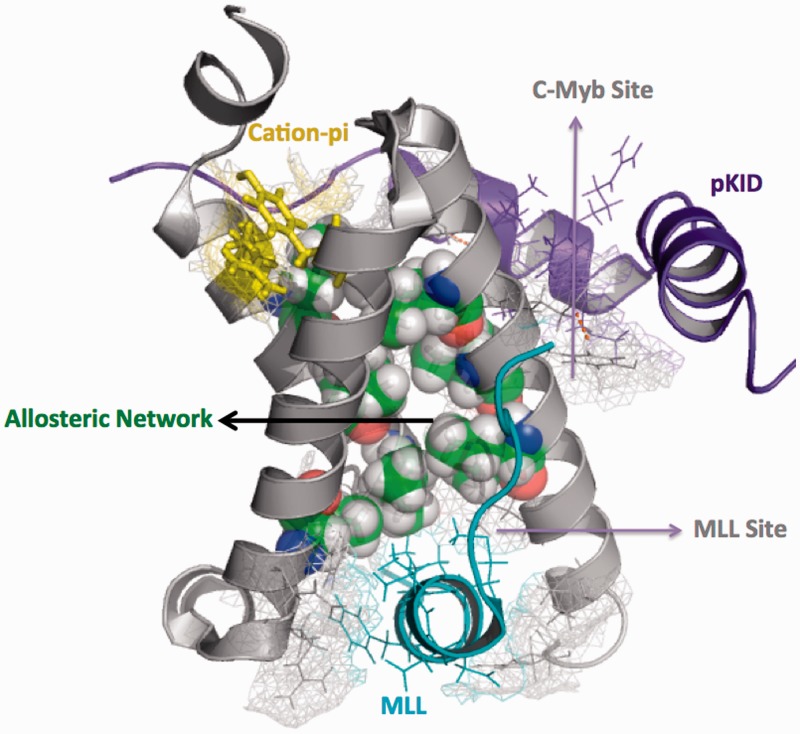
The allosteric network of contiguous hydrophobic side chains (atom colored spheres) within KIX domain (PDB ID 2LXT), connecting the two remote binding sites. Each binding site and the cation–pi bond are depicted in mesh representation. The two ligands and their respective TADs are shown in similar colors at their cognate KIX-binding surfaces.

The allosteric binding sites further endow KIX with the unique ability to communicate between these remote and isolated regions on its surface, providing a potential mechanism to modulate transcriptional activity through conformational transitions between bound and unbound states. Therefore, KIX has the potential to act as a powerful regulator of gene transcription through its allosteric binding sites, and plays a key role in transcriptional synergy. Biophysical characterization and quantification of allosteric communication pathways is intimately linked to protein dynamics and subtle rotameric re-adjustments at the atomic level. Although the high plasticity of KIX essentially adds to its function, enabling recognition of a diverse array of activator sequences, it also renders this domain intractable to crystallographic characterization, and thus NMR methods have mostly been used to understand the intricate details of conformational dynamics in KIX. Bruschweiler *et al.* (67,69) used NMR relaxation dispersion techniques and structural data to decipher the mechanism of allosteric transition in the MLL.KIX.pKID/c-Myb ternary complex. They monitored the conformational rearrangements within the KIX domain between the unbound (KIX alone), binary (KIX.MLL) and ternary (KIX.MLL.pKID) states and identified a non covalent contiguous network of residues connecting the two remote sites through which KIX communicates information about target presence at MLL site to the allosteric c-Myb/pKID site. Figure 4 depicts this hydrophobic network comprising of aliphatic side chains in the ternary KIX complex (PDB ID 2LXT) and it can clearly be seen that it connects the two spatially separate binding sites. This network was found to be conserved across yeast and mammalian KIX domains and was proposed to form a defined pathway for transmission of allosteric information by means of chemical shifts, residual anisotropic interactions and rotameric changes (67). The 90 residue spanning KIX domain has a non-uniform distribution of long chained and cyclic amino acids in its hydrophobic core, the interior being formed mainly by non aromatic residues as compared to the surface, which, in contrast, is enriched for aromatic residues. Further, the hydrophobic core displays greater conservation of aliphatic residues than for aromatic residues and it has been suggested that the observed evolutionary conservation of these aliphatic residues (namely *Leu-603*, *Leu-607*, *Ile-611*, *Leu-628*, *Leu-653*, *Ile-657* and *Ile-660*) is due to the fact that they constitute the allosteric regulatory network described above (69). Molecular dynamics (MD) studies further indicated that binding of MLL to KIX produced a conformational redistribution within the protein favoring binding of the second, remote ligand (55). The α1–α2 linker region and G2 helix of KIX have both been shown to play a key role in the conformational control (55,59). A detailed structural comparison of the binary and ternary KIX complexes showed that rigidity of KIX protein backbone was not significantly altered between the two states, but the methyl group nuclear overhauser effect patterns revealed a measurable rearrangement of the hydrophobic core and the residues involved in this small but significant repacking of the core were identified to be the same as those previously identified as part of the KIX allosteric network, listed above (67,69). Interestingly, this allosteric communication did not appear to be bi-directional in the initial studies, i.e. communication about presence of substrate at the c-Myb/pKID site to the MLL site could not be detected on account of standard MD timescale limitations, whereby only phenomena that take place on the scale of microseconds can be accessed, while others remain ‘invisible’. This limitation was recently overcome by a detailed MD study of allosteric communication in KIX employing enhanced sampling methods that allowed confirmation and visualization of the short-lived or invisible excited state of the ternary complex of MLL:KIX:pKID (70). The authors confirmed the experimental results by Bruschweiler *et al.* (67,69), providing a mechanistic model for the conformational transitions within the allosteric network, showing that the structure of the invisible state is similar to that of the MLL:KIX moiety in the ternary complex. Very recently, the first crystal structure of KIX domain has been reported by using a covalently linked small molecule ligand through tethering methods (71). This structure was then used to perform MD simulations for identification of specific side-chain motions that remodel KIX binding sites for mediating partner recognition through conformationally dynamic interfaces (71).

## KIX INHIBITION AND TRANSCRIPTIONAL SWITCHING

Unlike some coactivators that are specific to a single family of transcription factors, CBP/p300 binds to a plethora of targets and initiates gene activation at varied promoter sites, integrating several signal transduction pathways. For example, CBP/p300 also functions as a coactivator of NRs, and thus, despite being present in small amount and limiting concentration, CBP is at the epicenter of transduction events that lead to specific gene expression in the cell. Thus, there is huge competition for acquiring it, thereby entailing and even necessitating its precise regulation. In this context again, the KIX domain plays a central role for discriminating between various cellular signals by virtue of its binding promiscuity and allostery. The initial implications of two independent binding surfaces on KIX included selective use of these sites by transcription factors as both a mechanism to discriminate between inducible and constitutive activators and a mechanism for combinatorial recruitment of CBP by diverse TADs (54). In 2001, even before the second KIX-binding site and its synergistic regulatory effects were noticed, the existence of a unique KIX based transcriptional ‘switch’ was uncovered, that could toggle CBP from CREB-regulated to NR-regulated gene expression, wherein it was demonstrated that arginine specific methylation of CBP/p300 inhibits cAMP-dependent gene regulation (72). The authors found that CBP could smoothly switch partners by recruiting coactivator associated arginine methyltransferase 1 (CARM1), a type I protein arginine methyltransferase (PRMT), based on evidence that methylation could destabilize the structure of KIX domain.

Methylation is a common post-translational modification occurring mainly on arginine and lysine residues and PRMTs (of which 10 have been identified so far in mammals) methylate various proteins including histone and non histones (73). PRMTs have conventionally been classified into two classes based on their nature of arginine methylation, with type-I forming monomethylarginine and asymmetric dimethylarginine in contrast to type II PRMTs that form monomethylarginine and symmetric dimethylarginine (74–76). CARM1 is a type I PRMT, and it is known to methylate histone H3, CBP/p300, steroid receptor coactivator-3 and many other proteins (77–79).

In case of CBP/p300, the major site of methylation by CARM1 was shown to be *Arg-600* in the KIX domain, a residue discussed earlier in this review as being critical for KIX stabilization. Modification of the *Arg-600* was believed to destabilize the KIX domain by disrupting a buried cation–pi interaction between positively charged guanidinium group of this residue and the aromatic ring of *Tyr-640*, an interaction that is critical for specifying the unique cooperatively folded KIX structure as shown in Figure 2 (80). However, it is noteworthy that in a parallel study and in multiple other studies (both *in vitro* and *in vivo*) carried out by independent groups following the initial observation of KIX methylation at *Arg-600*, a consensus could not be reached for the exact region of methylation on CBP and it was argued that methylation by CARM1 actually occurs at an unstructured C-terminal extension of the CBP KIX domain (region 568–828), rather than its core region (63,81,82). Predominant methylation at this unstructured segment of KIX was hypothesized to affect the stability of CREB–CBP interaction, leading to diminished recognition (63). Despite the controversy of exact methylation site, it is quite clear that protein methylation at arginine residues within or near CBP-KIX domain is an important mechanism for signal transduction and CARM1 is critical for co-activator synergy in transcription. Thus, methylation of the CBP-KIX region (core or extended C terminal) by CARM1 can disrupt the CBP–CREB interaction and block transcription of CREB dependent genes, which in turn may render the limited CBP pool free and available for NR dependent gene expression (72,81,82). In this manner, KIX methylation effectively switches CBP to mediate NR-dependent gene transcription exclusively, and this switch is especially significant in view of the large number of cellular events that CREB is associated with, including glucose homeostasis, immune response and memory retention. Interestingly, the molecular switch concept opened up a completely new paradigm for interfering with gene regulation for medical purposes and led to new avenues of research concerning the reversibility of methylation based CREB–CBP inactivation, regulation of CARM1 itself, as well as the connection between methylation and ubiquitination of CBP, if any (83).

## KIX DOMAIN AS A PREFERRED TARGET FOR DRUG DEVELOPMENT

Small molecules that can interfere with the process of transcription can be very useful as therapeutic or co-therapeutic drugs. Moreover, such molecules can also be used as an important research tool in the study of biological processes and mechanisms. In the last decade, several small molecules have been found that can directly target protein-protein interactions involved in the process of transcription (84). Being an important domain in the transcriptional regulators like p300/CBP and MED15, KIX has been a preferred choice for researchers to identify or design small molecules with agonistic or antagonistic effect on it. Initially, protein grafting was used in combination with molecular evolution by phage display to identify phosphorylated miniature protein ligands that could bind to the CBP KIX with high nanomolar affinity (85). Within a year, in an NMR-based screening, a couple of compounds namely palmoic acid (KG-122) and naphthol AS-E-phosphate (KG-501), were found that could bind to CBP KIX domain (86). KG-122 binds to the MLL site of CBP KIX, but it alters the side chain conformation of *Lys-662*, which forms an ion pair with *Ser-133* phosphate of CREB. On the other hand, KG-501 docks in the c-Myb site of CBP KIX in the same manner as the KID domain. Thus, upon binding to the CBP KIX domain, both these compounds disturb the amino acids that are required for KID–KIX interaction, and so function as the inhibitors of CREB–CBP interaction attenuating the induction of cAMP responsive genes (86). Later, by using Renilla luciferase complementation assay, naphthol AS-E (unphosphorylated form of KG-501) was identified as the first high-affinity small molecule ligand of CBP-KIX and a cell-permeable strong inhibitor of KIX–KID interaction (87). The structure–activity relationship studies of naphthol AS-E by modifying the appendant phenyl ring, revealed preference for a small electron-withdrawing group at the para position to inhibit KIX–KID interaction (88). CREB not only regulates hepatic gluconeogenesis during fasting and in diabetes (89,90), but is also found to be over-expressed in different types of cancers (88,91). Thus, small molecules such as naphthol AS-E that can act as inhibitors of KIX–KID interaction are suitable structural templates for the development of potential drugs to benefit cancer and diabetic patients.

As mentioned previously, KIX domain of Gal11p/MED15 is targeted by AD of Pdr1p, a zinc cluster transcription factor, and its orthologs in yeast and fungi for up-regulation of multidrug resistance (2). Pathogenic fungi, especially *Candida* species including *C. glabrata*, account for up to 9% of all blood stream infections with crude mortality rate of 40% (92). So, in an attempt to propose novel antifungal therapy, as in the case of CREBP–CBP interaction, using a high thorough-put fluorescence polarization assay, few small molecular compounds like N16, K20 and I17 have been identified that can inhibit interaction between Pdr1p-AD and Gal11Ap-KIX of *C. glabrata* (93). Though these studies are still in infancy, such molecules have the potential to be modified for therapeutic or co-therapeutic purpose to check fungal infections in immunocompromised patients or patients undergoing chemotherapy.

## KIX IN PLANTS

As of date, KIX domains have not been characterized from plants, although sequence data have shown evidence for existence of both CBP-like proteins and MED15 subunit of mediator complex in various species of plants (21,94–96). However, research in this area is fast gaining pace with availability of an increasing amount of genomic and transcriptomic data, along with mutational studies from diverse plant species enabling reliable predictive analyses. Already, computational studies and protein family databases like the InterPro have revealed a conservation of CBP KIX domain sequence as well as predicted secondary structure in plants such as *Oryza sativa*, *Populu strichocarpa*, *Medicago truncatula*, *Glycine max* and several others, and these proteins are being identified as gene families in the plant kingdom (http://www.ebi.ac.uk/interpro/). Initial reports suggested that KIX domain may not be conserved in *Arabidopsis thaliana* but later studies using profile based search tools established the presence of at least five KIX-like domains in proteins of this plant including three HATs, namely HAT of CBP family 1 (HAC1), HAC5 and HAC12 (94,97,98). Examination of the domain architectures of these proteins revealed substantial diversification from the animal sequences, most notably the absence of bromodomains, suggesting evolutionary plasticity and potential modification of function, although critical interacting residues were conserved (21,94). Mutational studies on plant KIX containing proteins have revealed multiple developmental and phenotypic defects, and transcriptome analyses have suggested important roles for these domains in development (leaf maturation and floral transition) and stress responses (abiotic), in both monocot and dicots (20,99,100). Further, bioinformatics analyses have suggested a marked structural conservation of KIX domains in multiple paralogs of rice and *Arabidopsis* CBP/HAC proteins and MED15 subunits when compared with mouse CBP, yeast Gal11 KIX and human ARC105 KIX domains (21,95). Very recently, we performed an hidden markov model based identification of KIX domains from these two plants that revealed 11 proteins containing KIX domains each in rice and *Arabidopsis*, expanding the previously known repertoire. Intriguingly, in addition to CBP MED15 and RECQL5, KIX domain was found in F-box (FBX) proteins of rice. Although functional analysis is yet to be done, it seems that KIX domain in such FBX proteins can help them interact with the proteins targeted for proteasomal degradation. We also analyzed these proteins in terms of gene structure, domain architecture, phylogenetics, comparative transcriptomics and SNP based association analysis (21). This study provided several new insights including existence of proteins with tandem KIX domains, novel combinatorial arrangements and correlation of KIX with seed size related agronomic traits in rice. It remains to be seen whether the KIX gene families in plants have undergone sub-functionalization and neo-functionalization and, if so, it needs to be determined how and where the requisite changes have occurred in terms of sequence and structure. In summary, the plant kingdom offers a unique opportunity and enormous potential for research and exploration of the KIX domain.

## CONCLUSIONS

The KIX domain was discovered in p300/CBP as a three-helix bundle important for protein–protein interaction. This domain acts as docking site for ADs of several transcription factors including NRs. NRs and p300/CBP homologs are not encoded by yeast and fungal genomes, but NR-like ligand-activated transcription factors, which target KIX domain of MED15 for their transcriptional activities were recently characterized in yeast. MED15 is well-conserved mediator subunit found in all the eukaryotes ranging from fungi to animals and plants. Thus, transcription factors targeting KIX domain seem to be very ancient mechanism of transcriptional activation, predating divergence of fungi, metazoan and plants. This highlights very interesting evolutionary and functional aspects of KIX domain, which should be addressed by the scientific community. KIX domain is present across all the eukaryotic kingdoms ranging from yeast to animals and plants, but the proteins harboring this domain may not be conserved in all the eukaryotes. For instance, CBP is not found in fungi, and FBX KIX is reported only in three rice FBX proteins but not in FBX proteins of *Arabidopsis* and metazoans. We could not find any RecQL helicase with KIX domain in rice. In addition, the proteins that target KIX domains are not conserved in all the eukaryotes. NRs that target KIX domains of CBP and MED15 are found in metazoans but not in fungi and plants. Zinc cluster transcription factors that target MED15 KIX are specific to the kingdom of fungi. Adding to this complexity is the specificity of KIX–ligand interaction. There are many transcription factors that interact with MED15 KIX but not with the CBP KIX and *vice versa*. Thus, it is necessary to carry out a detailed in depth structural analysis of KIX domains in all these diverse class of proteins and their interaction with the cognate ligands, to help address the structural and functional convergence or divergence of this intriguing domain across different kingdoms. This understanding may also be very useful in designing different small molecules to target different specific KIX domains for clinical, therapeutic or agricultural applications. In addition to CBP, MED15 and RecQL proteins, recent discovery of KIX domain in three rice FBX proteins raises the possibility of presence of this domain in many other, heretofore unknown proteins, which may not have direct role in the assembly of transcriptional machinery. Future research programs involving structure-dependent computation algorithm may help in characterization of all the members of KIX domain containing protein family.

## FUNDING

Innovative Young Biotechnologist Award (IYBA) grant from the Department of Biotechnology (DBT), Government of India, awarded to [BT/BI/12/045/2008 to J.K.T. and BT/BI/12/040/2005 to G.Y.]; Research grant [BT/PR14519/BRB/10/869/2010 from DBT to J.K.T.]; Recipient of the junior research fellowship (JRF) of the University Grants Commission, Government of India (to A.V.). Funding for open access charge: National Institute of Plant Genome Research.

*Conflict of interest statement.* None declared.
